# Flexible Hydrophobic Paper-Based Microfluidic Field-Effect Biosensor Amplified by RNA-Cleaving DNAzyme-Based DNA Nanostructure for Mg^2+^ Detection

**DOI:** 10.3390/bios15070405

**Published:** 2025-06-24

**Authors:** Hui Wang, Yue He, Zhixue Yu, Ruipeng Chen, Zemeng Feng, Dongfei Chen, Waleid Mohamed El-Sayed Shakweer, Fan Zhang, Xuemei Nan, Mukaddas Mijit, Benhai Xiong, Liang Yang, Xiangfang Tang

**Affiliations:** 1 State Key Laboratory of Animal Nutrition and Feeding, Institute of Animal Science, Chinese Academy of Agricultural Sciences, Beijing 100193, China; wanghui10@caas.cn (H.W.); heyueh@163.com (Y.H.); 15831207256@163.com (Z.Y.); chen_ruipeng@yeah.net (R.C.); 18813015831@139.com (F.Z.); xuemeinan@126.com (X.N.); muukaddas@163.com (M.M.); xiongbenhai@caas.cn (B.X.); 2 Institute of Subtropical Agriculture, Chinese Academy of Sciences, Changsha 410125, China; fengzemeng@isa.ac.cn; 3 Graduate School of Biomedical Engineering, University of New South Wales, Sydney, NSW 2052, Australia; dongfei.chen2018@gmail.com; 4 Animal Production Department, Agricultural and Biological Research Institute, National Research Centre, 33 El-Buhouth Street, Dokki, Cairo P.O. Box 12622, Egypt; shakweer@gmail.com

**Keywords:** hydrophobic paper, similar DNA origami, microfluidic chip, electrochemical electrode, DNA nanoparticle, magnesium ion

## Abstract

Magnesium ions (Mg^2+^) play an important role in animal health, with their concentration in the bloodstream serving as a key indicator for hypomagnesemia diagnosis. In this study, a flexible hydrophobic paper-based microfluidic field-effect biosensor was developed for point-of-care Mg^2+^ detection, which integrated flexible hydrophobic paper, semiconducting single-walled carbon nanotubes (SWNTs) and a Mg^2+^-specific RNA-cleaving DNAzyme(RCD)-based DNA nanostructure. Flexible hydrophobic paper was synthesized by using cellulose paper and octadecyltrichlorosilane, improving mechanical strength and decreasing biological interference. To achieve high sensitivity, the Mg^2+^-specific RCD was functionalized with SWNTs, and then repeatedly self-assembled two different Y-shaped DNAs to construct a DNA nanostructure based on a similar DNA origami technique. This proposed biosensor exhibited a linear detection range from 1 μM to 1000 μM, with a detection limit of 0.57 μM, demonstrating its great stability, selectivity, and anti-interference performance. This innovative design offers promising potential for Mg^2+^ monitoring in real applications.

## 1. Introduction

The magnesium ion (Mg^2+^) is an essential divalent cation for cell survival and normal physiological metabolism, ranking just behind calcium, sodium, and potassium in importance [1,2,3,4]. Mg^2+^ shares several physiological functions with calcium [5], such as inhibiting nerve and muscle excitability, and plays a crucial role in the metabolism of carbohydrates, fats, and nucleic acids [6]. The Mg^2+^ concentration in animal blood is an important health indicator [7,8]. The reference serum magnesium levels for healthy animals are as follows: 0.74~0.95 mmol/L for cattle [9]. There are numerous methods available for measuring Mg^2+^ content, including complexometric titration [10], fluorescent sensors [11], and inductively coupled plasma emission spectroscopy [12]. However, these methods suffer from complicated operations, high equipment costs, and poor portability, rendering them unsuitable for real-time, on-site analysis. Therefore, a fast and portable method for blood Mg^2+^ detection is necessary to enable timely adjustments.

Flexible paper-based microfluidic field-effect sensors offer multiple advantages by integrating microfluidic platforms with semiconducting single-walled carbon nanotubes (SWNTs): (1) minimal sample and reagent use: they require very small volumes of samples and reagents [13]; (2) portability and simplicity: they eliminate the need for expensive laboratory infrastructure [14]; and (3) easy to fabricate and cost-effective: cellulose paper is abundant, inexpensive, and easy to process [15]. However, flexible paper-based sensors have several limitations, including low mechanical strength in aqueous solutions, biological interferences, and poor detection performance, including low sensitivity and a narrow dynamic range.

Octadecyltrichlorosilane (OTS) is a versatile organosilane widely used in surface modification due to its high-performance hydrophobicity, scalability, and environmental adaptability [16]. Its advantages include facile processing via scalable dip/spray-coating, chemical stability through covalent Si-O-Si bonding, UV-degradable and eco-friendly properties, cost-effectiveness compared to fluorinated alternatives, and enhanced water/oil barrier capabilities [17,18]. When covalently grafted onto cellulose paper, OTS improves mechanical durability and biological resistance, expanding its utility [19].

Our previously reported similar DNA origami [20,21] enables the precise design and fabrication of sophisticated DNA-based nanostructures, which are rationally engineered from DNA molecules [22]. Unlike conventional DNA origami [23,24,25], this method eliminates the need for a long, expensive single-stranded DNA scaffold. Instead, it employs multiple short oligonucleotides with specific base sequences that self-assemble in a programmed order to construct intricate DNA nanostructures.

In this study, a flexible paper-based microfluidic field-effect sensor was functionalized with an Mg^2+^-specific RNA-cleaving DNAzyme (RCD) for Mg^2+^ detection. To address mechanical durability and biological resistance challenges, cellulose paper was modified with polymerized OTS to fabricate flexible superhydrophobic paper. A cutting-and-pasting method was used to construct the microfluidic field-effect sensor. SWNTs are highly sensitive to the negatively charged materials, and the DNA nanostructure carries an inherent negative charge due to the presence of negatively charged phosphate groups. To overcome the detection performance limitations, Mg^2+^-specific RCDs were functionalized with SWNTs via amide bonds (-CONH-), which were further designed and assembled with a negatively charged DNA nanostructure to improve the sensitivity and dynamic range.

## 2. Materials and Methods

### 2.1. Chemical Reagent

Octadecyltrichlorosilane (OTS) (purity > 95%) was procured from the Sinopharm Chemical Reagent Co., Ltd. (Tianjing, China). Cellulose paper (Whatman No.5) was offered by Shanghai Jinpan Biotechnology Co., Ltd. (Shanghai, China). Conductive silver paste was acquired from Shenzhen Jingzhe Technology Co., Ltd. (Shenzhen, China). Pyrene carboxylic acid (PCA, 97%), Bovine Serum Albumin (BSA), sylgard 184 Silicone elastomer kits, and 1-(3-dimethylaminopropyl)-3-ethylcarbodiimide (EDC) were obtained from Sigma Aldrich (Beijing, China). A semiconducting single-walled carbon nanotube (SWNTs, 95%) was acquired from NanoIntegris Inc. (CA, USA). N-hexane (purity > 95%) was supplied by the Chongqing Chuandong Chemical Group Co., Ltd. (Chongqing, China). N, N-Dimethylformamide (DMF), n-hexane, and methanol were provided by Shanghai Mackin Biochemical Technology Co., Ltd. (Shanghai, China). All of the reagents were of analytical grade and were used as received without further purification. Ultrapure water produced by a Millipore Milli-Q (Beijing, China)system was used throughout the experiment.

The DNA oligonucleotides employed in this study were purchased from Shanghai Sangon Biotechnologies Co., Ltd. (Shanghai, China), with details provided in Table 1. All of the DNA oligonucleotides were dissolved in a 10 mM Tris-HCl solution (pH 7.4).

### 2.2. Apparatus

Surface morphology was characterized using a Hitachi Scanning Electron Microscope SU3500 (Hitachi High-Technologies, Tokyo, Japan). The water contact angle (WCA) was determined via a DataPhysics OCA 50AF contact angle goniometer (DataPhysics Instruments GmbH, Filderstadt, Germany) using the sessile drop method with a 5 μL droplet. Raman spectra were obtained using the Renishaw InVia Raman microscope (Renishaw plc, Wotton-under-Edge, UK) equipped with an imaging microscope (532 nm Diode and Ar ion lasers). Fourier transform infrared reflections were recorded using Nicolet IS10 (Thermo Fisher Scientific, Waltham, MA, USA). Electrochemical methods were collected by using the CHI 760E electrochemical workstation (CH Instruments, Shanghai, China).

### 2.3. Preparation of MFES/SWNTs-PCA

**Figure 1:**
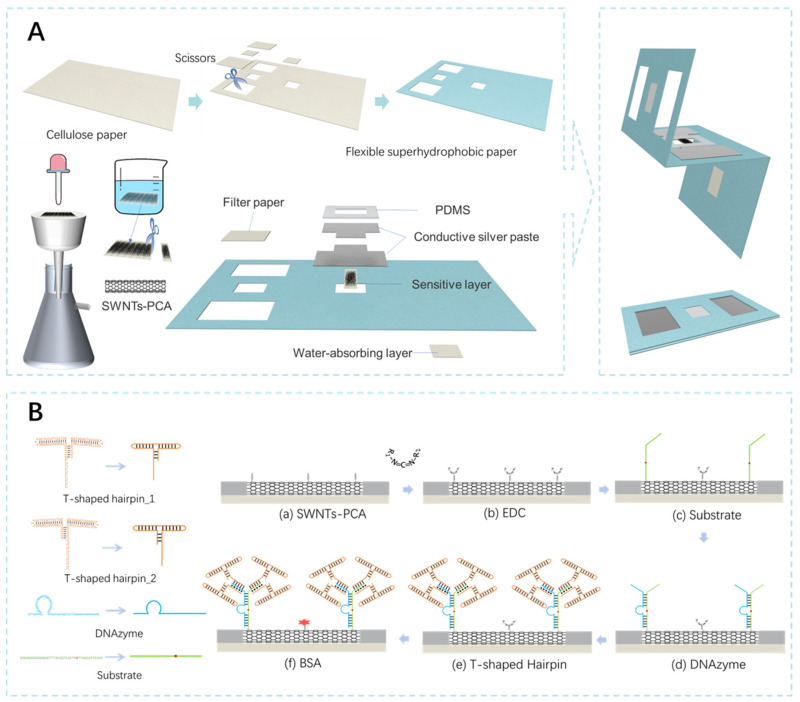
(**A**) Fabrication process of a flexible hydrophobic paper-based microfluidic field-effect sensor using the cutting and pasting method; (**B**) MFES/SWNTs-PCA functionalized with different chemical and biological materials successively (EDC, Substrate, DNAzyme, T-shaped hairpin_1, T-shaped hairpin_2 and BSA).

Figure 1A shows the fabrication process of a flexible hydrophobic paper-based microfluidic field-effect sensor (MFES/SWNTs-PCA) using the cutting-and-pasting method. Based on the design specifications, cellulose paper (60 mm × 36 mm) was cut into two sensitive holes (5 mm × 4 mm) and two electrode holes (12 mm × 10 mm) using scissors. Polymerized octadecyltrichlorosilane (Poly-OTS) was synthesized by mixing 0.5 mL of OTS with 10 mL of n-hexane and 20 μL of water, followed by ultrasonic treatment until it turned milky white. The cellulose paper was then immersed in Poly-OTS solution for 5 min, while the solution was gently shaken. After treatment, the paper was washed with hexane and ethanol to remove any residual material and was dried at room temperature.

Semiconducting ink was prepared by dispersing 0.1 mg of SWNTs powder dispersed in 15 mL of DMF, followed by ultrasonic treatment for 4 h. A long strip of cellulose paper was placed on a suction filtration device consisting of a filter and a vacuum pump, and the semiconducting ink was applied to retain the SWNTs on the surface of the cellulose paper. The filtered cellulose paper was then immersed in a 0.04 mg/mL PCA solution for 4 h. After treatment, the SWNTs-PCA decorated cellulose paper was washed multiple times with DMF and ethanol, then cut into a size of 8 mm × 2 mm in length and width. The sample filter paper, SWNTs-PCA strip, and bottom absorbent paper were pasted at the corresponding positions. Silver paste was screen-printed on the source and drain electrodes, and the junctions were sealed and blocked using PDMS.

### 2.4. Modification of MFES/SWNTs-PCA/RCD-Hairpin

Figure 1B shows the schematic diagram of MFES/SWNTs-PCA functionalized with different chemical and biological materials successively. Prior to biomaterial modification, MFES/SWNTs-PCA was activated by phosphate buffer (10 mM, pH 6.2) containing 10 mM EDC for 30 min and was then flushed with adequate 10 mM PBS to remove the residuals. The amine-labelled substrate was dropped on the surface of MFES/SWNTs-PCA overnight at 4 °C and was then hybridized with 50 μM of DNAzyme for 4 h at room temperature to form MFES/SWNTs-PCA/RCD. After that, MFES/SWNTs-PCA/RCD was incubated in 50 μM of T-shaped hairpin that was hybridized with the substrate and DNAzyme. Finally, the prepared device was immersed into 0.01 of mM BAS solution to prepare MFES/SWNTs-PCA/RCD-Hairpin.

### 2.5. Sensing Protocol

The resistance of the biosensor was calculated using Ohm’s law (R=U/I) at −0.1 V, and the relative resistance was calculated as the following equation:

$$Relative\;resistance=\left(R_{0}-R\right)/R_{0}\times 100\%$$

where $\;R_{0}$ represents initial resistance value, and R is the resistance after the exploration of the extracted solution.

## 3. Results

### 3.1. Characteristics of MFES/SWNTs-PCA

**Figure 2:**
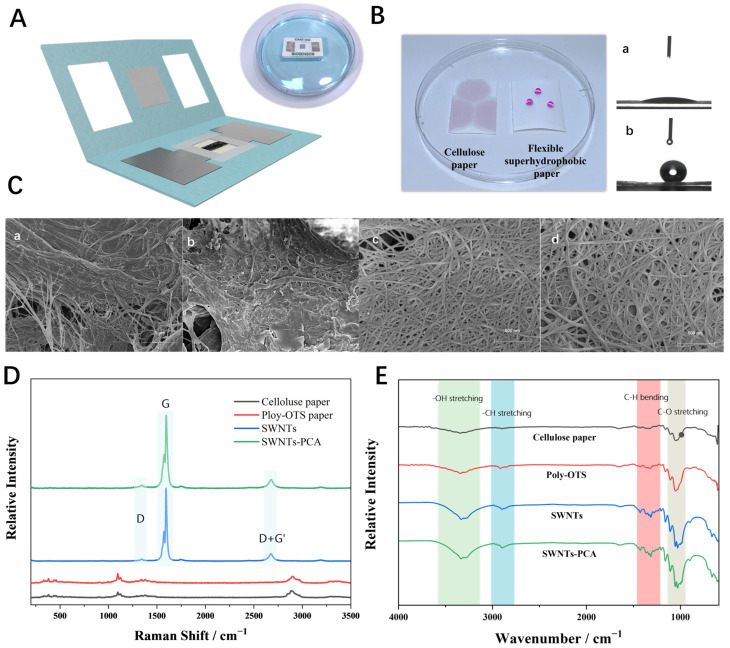
(**A**) Schematic diagram of MFES/SWNTs-PCA; (**B**) changes in water contact angles of cellulose paper (a) and cellulose paper after being functionalized with Poly-OTS (b); (**C**) SEM images, (**D**) Raman and (**E**) FTIR of cellulose paper (a), poly-OTS modified cellulose paper (b), MFES/SWNTs (c), and MFES/SWNTs-PCA (d).

Figure 2A shows MFES/SWNTs-PCA floating on the water surface, demonstrating the distinct hydrophilic and hydrophobic properties of the material. Figure 2B presents the water contact angles (WCA) of cellulose paper before and after poly-OTS treatment. The WCA of untreated cellulose paper was close to 0° due to the presence of hydrophilic groups, such as carboxyl and hydroxyl. After poly-OTS treatment, the WCA increased to 142.4°, indicating the successful decoration of cellulose paper with hydrophobic C18 alkyl chains. Figure S1 shows the relationship between the water contact and incubation time of cellulose paper functionalized with poly-OTS, presenting a positive correlation. The WCA reached a maximum of 144.3° at 9 min, indicating optimal hydrophobicity, which is ideal for preparing the hydrophobic regions of MFES. Figure 2C shows the surface morphology investigated by scanning electron microscopy (SEM). Cellulose paper had a rough surface with fibers of different shapes and diameters. After superhydrophobic solution treatment, the overall diameter of the fibers becomes thicker; meanwhile, many smooth flake-like structures existed on the surface, indicating OTS formed the micro-to-nanoscale structure [16]. SWNTs before and after modified with PCA in (c) and (d) manifest the high-density network structure on the surface [26]. However, there is no significant difference between SWNTs and SWNTs-PCA because of the small molecule of PCA.

Raman spectroscopy analysis is a technique used to study the molecular structure and vibrational modes of materials. In Figure 2D, several small absorption peaks were observed in the Raman spectrum of cellulose paper. After modification with poly-OTS, these absorption peaks increased significantly, especially at 1045 cm^−1^ (C-O stretching vibration), 1098 cm^−1^ (C-H stretching vibration), 2852 cm^−1^ (C-H stretching vibration), and 3329 cm^−1^ (H stretching vibration), which are consistent with the characteristic peaks of poly-OTS. For SWNTs, the characteristic bands were located at 1344 cm^−1^ (D band), 1598 cm^−1^ (G band), and 2678 cm^−1^ (D+G’ band). When PCA was fixed on the surface of the SWNTs, the G band shifted to the higher frequency, and the relative intensities of the three bands increased slightly, indicating the successful attachment of PCA to SWNTs.

In Figure 2E, the pristine cellulose paper exhibits characteristic broad O-H stretching vibrations at 3400–3200 cm^−1^ and strong C-O stretching peaks at 1160 cm^−1^ and 1050 cm^−1^. After Poly-OTS modification, significant spectral changes occurred: new prominent C-H stretching peaks emerged at 2920 cm^−1^ and 2850 cm^−1^ due to the salinization reaction, corresponding to the long alkyl chains of OTS. Compared with Poly-OTS modified cellulose paper, both SWNTs and SWNTs-PCA exhibited enhanced vibrational modes at 600, 652, 1032, and 1052 cm^−1^. The SWNTs-PCA spectrum additionally exhibited a marginally intensified peak at 3335 cm^−1^, potentially attributable to carboxyl group hydrogen-bonding vibrations.

### 3.2. Characteristics of MFES/SWNTs-PCA/RCD-Hairpin

#### 3.2.1. Electrochemical Characteristics

**Figure 3:**
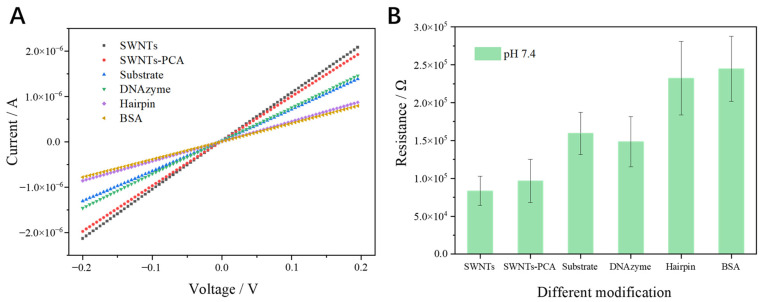
(**A**) Linear sweep voltammetry and (**B**) resistances of MFES/SWNTs modified with different materials (PCA, substrate, DNAzyme, T-shaped Hairpin, and BSA); each data point was an average of measurements from 3 independent biosensors.

Linear sweep voltammetry is an effective method for characterizing the resistance of SWNTs modified by different chemical/biological materials. Each curve in Figure 3A shows a linear relationship between current and voltage, with the slopes decreasing consistently across the modifications. Using Ohm’s law, the resistance values at −0.1 V were calculated and shown in Figure 3B. The resistance of unmodified SWNTs was 82.97 KΩ. When modified with PCA, the resistance slightly increased to 96.04 KΩ, indicating the successful attachment of PCA to SWNTs via C-C bonding [27]. Upon covalent bonding of the NH_2_ labelled substrate to SWNTs-PCA, the resistance further increased to 160.11 KΩ. Interestingly, when DNAzyme hybridized with the substrate to form RCD, the resistance decreased. This is likely due to the single-stranded DNA detaching from the SWNTs’ surface, where the higher negative charge of ssDNA compared to the RCD contributed to this change. Further fictionization with T-shaped hairpin and BSA caused a substantial increase in resistance. This increase is attributed to the strong electronegativity of these materials, which reduced the number of holes in the p-SWNTs, thereby increasing resistance [28,29].

#### 3.2.2. Optimization

Figure S2A shows MFES/SWNTs-PCA/RCD-Hairpin employed to detect two different Mg^2+^ concentrations (10 μM and 1000 μM) in a pH 7.4 Tris-HCl buffer solution with varying incubation times. It was clear that the relative resistance of MFES/SWNTs-PCA/RCD-Hairpin was not only proportional to Mg^2+^ concentrations but also incubation time. Within 20 min, the relative resistance was increasing with incubation time. However, the growth rate of relative resistance slowed down significantly. To reduce the detection time, 20 min was selected as the optimal incubation time for the experiment.

In Figure S2B, the *I_DS_-V_DS_* plot illustrates the non-linear relationship between current and voltage in the range of −0.2 V to 0.2 V for MFES/SWNTs-PCA/RCD-Hairpin exposure to different Mg^2+^ concentrations. The main reason may be attributed to the semiconducting properties of single-walled carbon nanotubes, which are influenced by the alkaline buffer solution approximately equal to the negative bias voltage. Additionally, at voltages above 0 V, the current may exhibit peaks due to the reduction reaction of silver oxide, indicating the presence of unsealed silver paste in the organic silicone gel. The resistances at each voltage were calculated using Ohm’s law as shown in Figure S2C. The resistance values are roughly equivalent to the relative resistances in Figure S2D when the voltage is below −0.02 V. To mitigate this effect, a voltage of −0.1 V was chosen in subsequent experiments to simplify the detection process. The optimal incubation time and detection voltage were determined to be 20 min and −0.1 V, as discussed in Figure S2 in the supporting information. These conditions were used in subsequent experiments to streamline the detection process.

#### 3.2.3. Performance Evaluation

**Figure 4:**
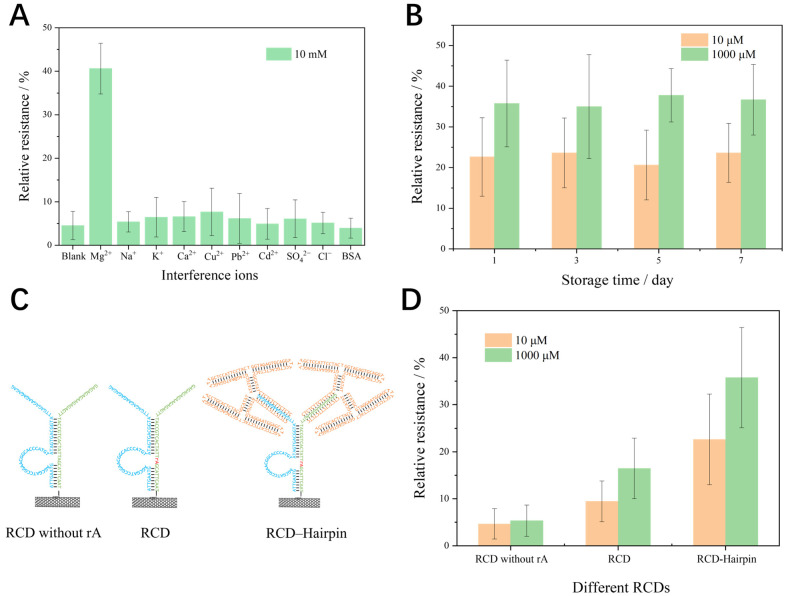
(**A**) Relative resistances of MFES/SWNTs-PCA/RCD-Hairpin before and after exposure to 10 mM inference ions (Na^+^, K^+^, Ca^2+^, Mg^2+^, Zn^2+^, Pb^2+^, Cd^2+^, SO_4_^2−^, and Cl^−^); (**B**) changes in relative resistance of MFES/SWNTs-PCA/RCD-Hairpin over time, illustrating the storage stability; (**C**) structure details of RCD without rA, RCD, and RCD-Hairpin; (**D**) relative resistances influenced by the presence of the ‘rA’ site and DNA structure for 10 μM and 1000 μM concentrations. (Each data point was an average of measurements from 3 independent biosensors.).

To assess the sensor selectivity, the MFES/SWNTs-PCA/RCD-Hairpin sensor was exposed to various 10 mM metal ions and BSA that might be present in real samples. As shown in Figure 4A, the relative resistance for 10 mM Mg^2+^ was approximately 28.6. In comparison, the relative resistances for other metal ions were significantly lower and hovered near the blank Mg^2+^ detection results, indicating minimal interference from these ions. These results indicated that the MFES/SWNTs-PCA/RCD-Hairpin sensor exhibits high selectivity for Mg^2+^.

A storage stability assessment was performed to evaluate the biosensor’s preservation capability under controlled environmental conditions. MFES/SWNTs-PCA/RCD-Hairpins were fabricated and stored at 4 °C, with measurements taken at different intervals to detect two different Mg^2+^ concentrations. The results in Figure 4B indicated that the relative resistance remained consistent over time, demonstrating that the sensor performance is stable during prolonged storage. These findings indicate that MFES/SWNTs-PCA/RCD-Hairpins maintain excellent stability for extended periods when stored in a refrigerator at 4 °C.

The sensitivity of RCD-based semiconducting biosensors depends on the RNA cleavage site and the DNA-sequence length. Several RCD-based semiconducting biosensors, as shown in Figure 4C, were designed and tested at two different Mg^2+^ concentrations. The results in Figure 4D indicate that, compared to MFES/SWNTs-PCA/RCD, the relative resistances of MFES/SWNTs-PCA/RCD without the ‘rA’ site remained largely unchanged after exposure to Mg^2+^. In contrast, the relative resistances of MFES/SWNTs-PCA/RCD-Hairpin were significantly higher. The sensing mechanism of the semiconducting SWNTs-based biosensor originates from resistance changes induced by the electronegativity of biomaterials. Due to the inherent negative charge of DNA, both the RNA cleavage site and sequence length can alter the quantity of DNA on the surface of semiconducting SWCNTs. Therefore, while maintaining an identical ‘rA’ site of MFES/SWNTs-PCA/RCD, increasing the number of bases in DNA on semiconducting SWNT-based biosensors can enhance detection sensitivity.

### 3.3. Sensitivity Amplified Using Similar DNA Origami Method

Based on the aforementioned analysis, it is obvious that the length of the bio-electronegativity DNA sequence is proportional to the sensitivity of the DNA-based semiconducting biosensor. However, the cost of commercially synthesized long-chain DNA (>150 bp) is relatively high, which limits the broader adoption and application of DNA-based semiconducting biosensors.

**Figure 5:**
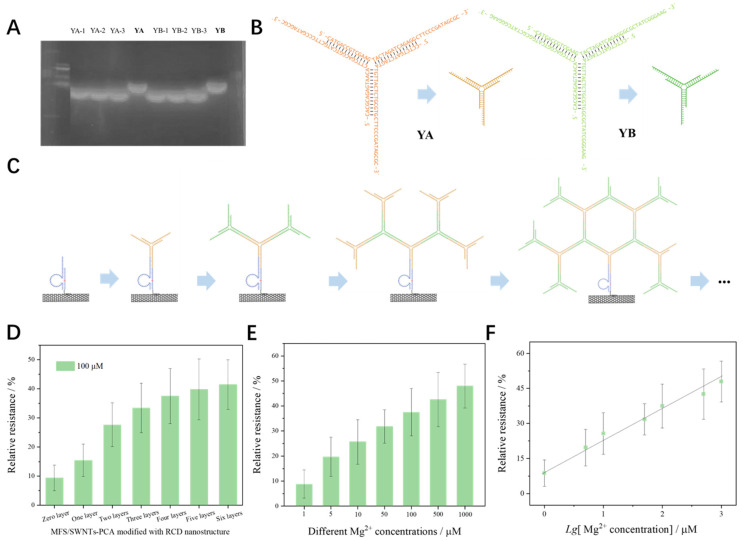
(**A**) Electropherograms of various single-stranded DNAs and Y-shaped DNA structures; (**B**) secondary structure and schematic diagram of YA and YB; (**C**) different MFES/SWNTs-PCARCD-Nanostructure constructed with DNAzyme, YA, and YB by layer-by-layer method; (**D**) relative resistances of MFES/s-SWNTs functionalized with different RCD-Nanostructures upon exposure to 100 mM Mg^2+^; (**E**) relative resistances of the optimal MFES/SWNTs-PCA/RCD-Nanostructure at different Mg^2+^ concentrations; (**F**) linear regression analysis for Mg^2+^ concentration detection. (Each data point was an average of measurements from 3 independent biosensors.).

To cut down the cost, we proposed a similar DNA origami technique using short single-stranded DNAs to construct complex DNA nanostructures. Table S1 lists the redesigned substrate, DNAzyme, and six different single-stranded DNAs. The Y-shaped structures, YA and YB, were synthesized through the hybridization of three complementary single-stranded DNAs, with the corresponding electrophoresis image in Figure 5A. Figure 5B shows the structural schematic diagrams of YA and YB. Based on the prepared MFES/SWNTs-PCA/RCD, the MFES/SWNTs-PCA/RCD-Nanostructure in Figure 5C was prepared by using the layer-by-layer self-assembly process, with each step maintained at room temperature for 2 h.

Figure 5D shows the relative resistances of MFES/SWNTs-PCA/RCD-Nanostructures with varying layers used to measure 100 μM Mg^2+^. After considering the preparation complexity and the sensitivity improvement, the MFES/SWNTs-PCA/RCD-Nanostructure with five layers was selected for subsequent experiments.

In Figure 5E, the optimized MFES/SWNTs-PCA/RCD-Nanostructure was employed to measure Mg^2+^ concentrations ranging from 1 μM to 1000 μM. The results indicate a linear relationship between the relative resistance and the logarithm of the Mg^2+^ concentrations. The regression equation, as shown in Figure 5F, is $\;y=12.485{log}_{10}\left({Mg}^{2+}\;concentration\right)+10.811\left(R^{2}=0.9852\right).$ The limit of detection is 0.57 μM.

Compared to many reported Mg^2+^ sensors, shown in Table 2, which primarily rely on fluorescence or colorimetric probes, the MFES/SWNTs-PCA/RCD-Nanostructure offers significant advantages. Conventional fluorescence- and color-based sensors often suffer from a narrow linear range and high detection limits. In contrast, the electrochemical biosensing approach of the MFES/SWNTs-PCA/RCD-Nanostructure provides superior detection performance, featuring a wide linear range and a lower detection limit.

### 3.4. Real Sample Analysis

To evaluate the practicality of the proposed sensor, the MFES/SWNTs-PCA/RCD-Nanostructure was applied to measure Mg^2+^ concentrations in blood samples from dairy cows. The blood samples were collected from the veins of dairy cows in southeastern Beijing and were filtered through a 0.22 μm membrane to remove hemoglobin and macromolecular proteins. The filtered liquid was then mixed with a pH 7.4 buffer solution and was directly applied to the MFES/SWNTs-PCA/RCD-Nanostructure for measurement. In addition, the filer samples were analyzed using an atomic absorption spectrometer (AAS) for comparison. The detection results, as shown in Table 3, indicated a recovery ranging from 92.54% to 107.72%. The recovery error was within 10%, with a *p*-value from the T-test (*p* > 0.05), suggesting no significant difference between the methods. These results demonstrated that the MFES/SWNTs-PCA/RCD-Nanostructure is capable of accurately determining Mg^2+^ concentrations in real samples with high accuracy and reliability.

## 4. Conclusions

In this study, a flexible hydrophobic paper-based microfluidic field-effect sensor was developed by using the cutting-and-pasting method and it was then decorated with an RNA-cleaving DNAzyme-based DNA nanostructure for the detection of Mg^2+^ levels. A variety of new technologies have significantly enhanced the performance of biosensors. Poly-OTS was synthesized and then modified with cellulose paper as was the hydrophobic region, with a maximum water contact angle of 142.4°. This improves the mechanical strength in aqueous solutions and biological anti-interference ability. Moreover, DNA nanostructure constructed by using a similar DNA origami technique can reduce the preparation cost and improve sensitivity. The optimized biosensor presented a wide linear range from 1 μM to 1000 μM, with a detection limit of 0.57 μM. This simple, robust, and selective biosensor offers accurate results even in real samples, providing a detection option for Mg^2+^.

## Figures and Tables

**Table 1:** DNA oligonucleotides used in this study.

Name	Sequence and Modifications (from 5′-3′)
Substrate with rA	GAGAGAGAAGAGAGTTTACCGTCACTATT/rA/GCATTCAAT-(CH_2_)_6_-NH_2_
Substrate without rA	GAGAGAGAAGAGAGTT TACCGTCACTATTAGCATTCAAT-(CH_2_)_6_-NH_2_
DNAzyme	ATTGAATGAGCGATCCGGAACGGCACCCATGTATAGTGACGGTATTGAGAGAAGAGAGAG
T-shaped hairpin_1	ATATGCGTAGGAATGGAGCTTTGCTCCATTCCTACTGGTAGAGTGCAGGTTTCCTGCACTCTACC GCATATCTCTCTTCTCTCTC
T-shaped hairpin_2	CTCTCTTCTCTCTCATATGCGTAGGAATGGAGCTTTGCTCCATTCCTACTGGTAGAGTGCAGGTTTCCTGCACTCTACC GCATAT

**Table 2:** Compare the detecting performances of MFES/SWNTs-PCA/RCD-Nanostructure with other sensors.

Sensor	Method	Linear Range (μM)	Detection Limit (μM)	Reference
NHMI-Mg^2+^	Fluorescence	0.25~2.5	0.069	[30]
NCDs-Mg^2+^	Fluorescence	80~720	60	[31]
PtOEP/9-AEC	Fluorescence	5~70	2.52	[32]
SBOD	Fluorescent	10~60	3.8	[33]
L-tryptophan-AgNPs sensor	Colorimetric probe	1~200	3	[34]
DNAzyme-CdTe QDs)	Fluorescence	0.001~0.020	0.0003	[35]
ECL Biosensor	Electrochemi-luminescence	10~10,000	2.8	[36]
FHBS-Biosensor	Fluorescence	0.4~70	0.4	[37]
SB-Mg^2+^ Biosensor	Fluorescence	-	7.1	[38]
MFES/SWNTs-PCA/RCD-Nanostructure	IV	1~1000	0.57	This work

**Table 3:** Mg^2+^ concentrations in actual samples determined by the proposed biosensor and AAS.

Sample	Added Mg^2+^ Concentration (mg/L)	The Proposed Biosensor (mg/L)	AAS (mg/L)	Recovery (%)
1	-	15.76	14.78	106.63
	50	69.78	-	107.72
2	-	16.87	18.23	92.54
	50	71.42	-	104.68
3	-	14.12	13.34	105.58
	50	67.31	-	106.23

## Data Availability

Data are contained within the article and supplementary materials.

## Supplementary Materials

The following supporting information can be downloaded at: https://www.mdpi.com/article/10.3390/bios15070405/s1. Figure S1. Water contact angle affected by the incubation time of cellulose paper modified with poly-OTS; Figure S2. (A) *I_DS_-V_DS_* plot of MFS/SWNTs-PCA/RCD-Hairpin exposure to different Mg^2+^ concentrations; (B) Resistances at each voltage after MFS/SWNTs-PCA/RCD-Hairpin exposure to different Mg^2+^ concentrations; (C) Relative resistances of MFS/SWNTs-PCA/RCD-Hairpins exposure to different Mg^2+^ concentrations; (D) Relative resistance of MFS/SWNTs-PCA/RCD-Hairpins changing with the voltage for different Mg^2+^ concentration; Each data point is an average of measurements from 3 independent biosensors; Figure S3. (A) Linear relationship between Mg^2+^ concentrations and relative resistance of MFES/SWNTs-PCA/RCD-Hairpin; (B) Linear regression of the relative resistance and the logarithm of the Mg^2+^ concentration; Table S1. DNA oligonucleotides for RCD-Nanotree.
