# Supplementary material for: Flexible Hydrophobic Paper-Based Microfluidic Field-Effect Biosensor Amplified by RNA-Cleaving DNAzyme-Based DNA Nanostructure for Mg2+ Detection
Source: Biosensors (Basel). 2025 Jun 24;15(7):405. doi: 10.3390/bios15070405 (PMC12293950; doi:10.3390/bios15070405)
Supplement: Supplementary file 1 [file biosensors-15-00405-s001.zip › biosensors-3658489-supplementary.pdf]

Supporting Information

Flexible Hydrophobic Paper-Based Microfluidic Field-Effect Biosensor Amplified by RNA-Cleaving DNAzyme-Based DNA Nanostructure for Mg^{2+} Detection

Hui Wang [1,†], Yue He [1,†], Zhixue Yu [1], Ruipeng Chen [1], Zemeng Feng [2], Dongfei Chen [3], Waleid Mohamed El-Sayed Shakweer [4], Fan Zhang [1], Xuemei Nan [1], Mukaddas Mijit [1], Benhai Xiong [1], Liang Yang [1,*] and Xiangfang Tang [1,*]

1 State Key Laboratory of Animal Nutrition and Feeding, Institute of Animal Science, Chinese Academy of Agricultural Sciences, Beijing 100193, China

2 Institute of Subtropical Agriculture, Chinese Academy of Sciences, Changsha 410125, China

3 Graduate School of Biomedical Engineering, University of New South Wales, Sydney, NSW 2052, Australia

4 Animal Production Department, Agricultural and Biological Research Institute, National Research Centre, 33 El-Buhouth Street, Dokki, Cairo P.O. Box 12622, Egypt

* Correspondence: yangliang@caas.cn (L.Y.); tangxiangfang@caas.cn (X.T.)

† These authors contributed equally to this work

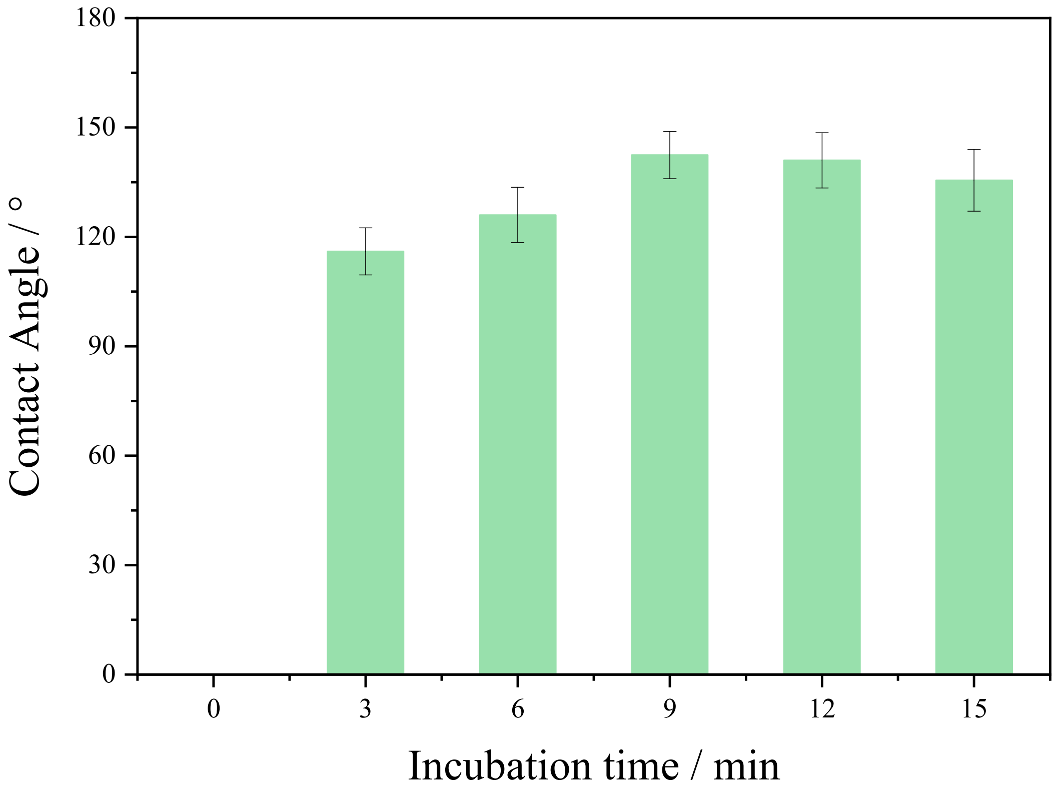

Figure S1. Water contact angle affected by the incubation time of cellulose paper modified with poly-OTS. (Each data point was an average of measurements from 3 independent biosensors.)

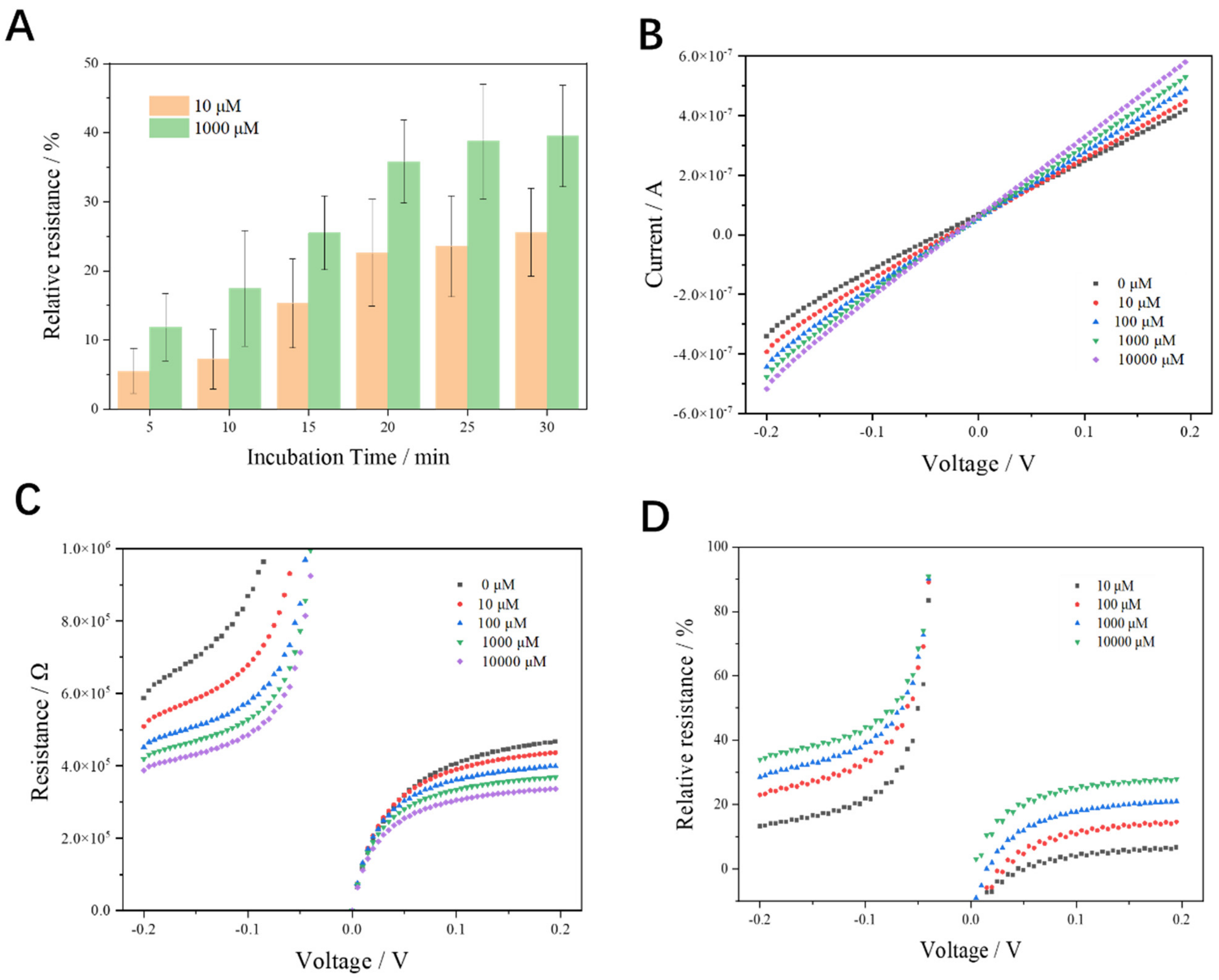

Figure S2. *(A)* I_{DS}*-*V_{DS} *plot of MFS/SWNTs-PCA/RCD-Hairpin exposure to different* Mg^{2+} *concentrations; (B) resistances at each voltage after MFS/SWNTs-PCA/RCD-Hairpin exposure to different* Mg^{2+} *concentrations; (C) relative resistances of MFS/SWNTs-PCA/RCD-Hairpins' exposure to different* Mg^{2+} *concentrations; (D)relative resistance of MFS/SWNTs-PCA/RCD-Hairpins changing with the voltage for different* Mg^{2+} *concentration; each data point is an average of measurements from 3 independent biosensors.*

Linear relationship

The linear relationship was studied by using MFES/SWNTs-PCA/RCD-Hairpin to measure the different Mg^{2+} concentrations under the optimized parameters. The results are shown in Figure S3. It was obvious that the relative resistance was proportional to the Mg^{2+} concentrations in the range from 1 μM to 10 mM, but the growth rate decreased for the high Mg^{2+} concentration. After the data analysis, the relative resistance revealed two linear relationships with the logarithm of the Mg^{2+} concentration. In the low Mg^{2+} concentration range from 1 μM to 100 μM, the regression equation was $Relative\ resistance=11.282\log_{10}(Mg^{2+}\ concentration)\ +8.74$ with a correlation coefficient of 0.9778. On the other

hand, in the high Mg^{2+} concentration range from 100 μM to 10 mM, the regression equation was *Relative resistance=4.6744log₁₀(Mg²⁺ concentration) +21.32* with a correlation coefficient of 0.9741. The detection limit of MFES/SWNTs-PCA/RCD-Hairpin was estimated to be 0.65 μM (N/S=3), which can meet the measurement requirements of a real sample.

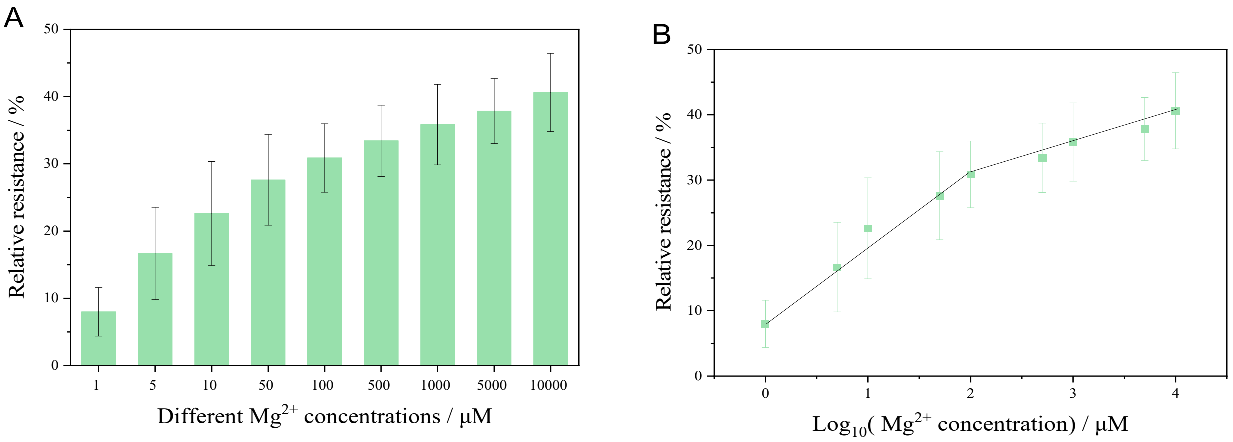

Figure S3. *(A)Linear relationship between Mg^{2+} concentrations and relative resistance of MFES/SWNTs-PCA/RCD-Hairpin; (B) linear regression of the relative resistance and the logarithm of the Mg^{2+} concentration. (Each data point was an average of measurements from 3 independent biosensors.)*

Table S1. DNA oligonucleotides for RCD-Nanotree.

Name	Sequence and modifications (from 5' -terminus)
Substrate	ACCGTCACTATT/rA/ GCATTCAAT-$(CH_2)_6$-NH_2
DNAzyme	ATTGAATGAGCGATCCGGAACGGCACCCATGTATAGTGACGGTATT GCGCTATCGGGAAG
YA-1	CACGCAGAGTAACACATGACCGTCGAAGCTTCCCGATAGCGC
YA-2	CTTCGACGGTCATGTACTAGATCAGAGGCTTCCCGATAGCGC
YA-3	CCTCTGATCTAGTATGTTACTCTGCGTGCTTCCCGATAGCGC
YB-1	CACGCAGAGTAACACATGACCGTCGAAGGCGCTATCGGGAAG
YB-2	CTTCGACGGTCATGTACTAGATCAGAGGGCGCTATCGGGAAG
YB-3	CCTCTGATCTAGTATGTTACTCTGCGTGGCGCTATCGGGAAG
